# A System of Operative Surgery

**Published:** 1909-09

**Authors:** 


**A System of Operative Surgery.** By Various Authors. Edited by F. F. Burghard, M.S., F.R.C.S. Vol. I. Pp. xxxi, 751. London : Oxford Medical Publications. 1909.

This handsome and copiously illustrated volume proves decidedly disappointing on perusal. Its 736 pages contain so little that is not better put in other text-books, that it is difficult to escape the conclusion that the book has been written to order, and not because its authors have anything fresh to say or any enthusiasm in saying it.

Mr. Lockwood opens the work by an article on general principles. The style of this is very casual, and its scope very incomplete. A sensible combination of aseptic and antiseptic methods is recommended which will appeal to all whose main object is good results rather than the upholding of a strict system. The retention of marine sponges for surgical use, and the elaborate method of their cleansing, is a relic of antiquity which we should not have expected in a modern treatise. The use of face masks only when the surgeon is suffering from a cold or has to speak seems to indicate a reluctance to adopt an obvious and valuable precaution.

Captain J. W. Houghton give a very useful account of the methods of local and spinal anæsthesia, which is practically a résumé of Mr. Barker's work on this subject. The value of this section would have been greatly increased if the indications for the employment of these forms of anæsthesia had been more thoroughly discussed, and the question of dangers and mortality of spinal anæsthesia definitely considered.

The great bulk of the volume, dealing with amputations, operations on joints, muscles, nerves and tendons, is by the pen of Mr. Burghard himself, and contains a clear description of ordinary operative measures with a commendable exclusion of obsolete and useless procedures. There is no mention of the amputation of the thigh together with part of the os innominatum, an operation which may well claim a definite recognition since the method of abolition of shock by spinal anæsthesia has been introduced. Neither is there any reference to the method of control of hemorrage in amputation of the thigh by pressure on the common iliac artery through an abdominal incision. The suture of arteries is dealt with in a very partial manner, the work of Carrel and his pupils, which forms the basis of all our knowledge of this subject, being scarcely mentioned. There is an excellent description of Matas's operations for aneurism, supplemented by recent information from the originator of these methods.

In many places the account of anatomical points is deficient or misleading. For example, the channels of collateral circulation after ligature of the main vessels are never given, the left iliac artery is said to be crossed by the rectum, and to be covered by the meso-rectum, and the nerve supply of the pyriformis and obturators is given as the inferior gluteal. The account of nerve suture contains no adequate discussion of the results to be expected from the primary and secondary operations nor any recognition of the different behaviour of the nerves of protopathic and epicritic sensation.

An excellent account of the methods of removal of the gasserian ganglion is somewhat marred by the wretched and useless illustrations. And there is no mention of the method of attacking the trigeminal nerve roots by deep injections of alcohol.

The account of tendon grafting and of the operative treatment of fractures are clear and up to date.

The separation of operations on the joints into those for nontuberculous and those for tuberculous affections appear to us to be very cumbrous, and must necessitate a great amount of repetition. For example, no less than two pages are devoted to a description of the removal of the non-tuberculous synovial membrane of the knee-joint. All this will have to be repeated in the next volume.

A remarkable statement is made relating to the treatment of displaced knee cartilages to the effect that after the first attack immediate massage and movements should be applied. Surely this is just the way to convert a torn cartilage into a loose cartilage; and Sir William Bennett, who is such an extreme advocate for early massage and movements in all other conditions, has emphasised the necessity of the first attack of cartilage displacement being treated by prolonged immobilisation.

The article on plastic surgery, by Mr. Legg, affords but little ground for comment. The pictures in this section are a model of clearness.

In many parts of the book, however, the clearness and usefulness of the pictures has been sacrificed to their mere number and artistic finish. Those illustrating the ligature of arteries of the head and neck are particularly useless, because the actual field of operation shown is so small, and the details are so indistinct.

The word thyroid is spelt thyreoid throughout the book, and this is an innovation which is as ugly as it is unwarranted in these days when the tendency is towards phonetic simplicity in orthography.

